# Erythropoietin Protects Adult Retinal Ganglion Cells against NMDA-, Trophic Factor Withdrawal-, and TNF-α-Induced Damage

**DOI:** 10.1371/journal.pone.0055291

**Published:** 2013-01-30

**Authors:** Zhi-Yang Chang, Ming-Kung Yeh, Chiao-Hsi Chiang, Yi-Hao Chen, Da-Wen Lu

**Affiliations:** 1 Graduate Institute of Life Sciences, National Defense Medical Center, Neihu, Taipei, Taiwan; 2 Institute of Preventive Medicine, National Defense Medical Center, Sanhsia, Taipei, Taiwan; 3 School of Pharmacy, National Defense Medical Center, Neihu, Taipei, Taiwan; 4 Department of Ophthalmology, Tri-Service General Hospital, National Defense Medical Center, Neihu, Taipei, Taiwan; The University of Hong Kong, Hong Kong

## Abstract

**Purpose:**

This study aimed to evaluate the neuroprotective effect of EPO in the presence of *N*-methyl-d-aspartate (NMDA)-, trophic factor withdrawal (TFW)-, and tumor necrosis factor-alpha (TNF-α)-induced toxicity on total, small, and large retinal ganglion cells (RGCs).

**Methods:**

Retinal cells from adult rats were cultured in a medium containing brain-derived neurotrophic factor (BDNF), ciliary neurotrophic factor (CNTF), basic fibroblast growth factor (bFGF), and forskolin. Expression of RGC markers and EPOR was examined using immunocytochemistry. RGCs were classified according to their morphological properties. Cytotoxicity was induced by NMDA, TFW, or TNF-α. RGC survival was assessed by counting thy-1 and neurofilament-l double-positive cells.

**Results:**

EPO offered dose-dependent (EC_50_ = 5.7 ng/mL) protection against NMDA toxicity for small RGCs; protection was not significant for large RGCs. Time-course analysis showed that the presence of EPO either before or after NMDA exposure gave effective protection. For both small and large RGCs undergoing trophic factor withdrawal, EPO at concentrations of 1, 10, or 100 ng/mL improved survival. However, EPO had to be administered soon after the onset of injury to provide effective protection. For TNF-α-induced toxicity, survival of small RGCs was seen only for the highest examined concentration (100 ng/mL) of EPO, whereas large RGCs were protected at concentrations of 1, 10, or 100 ng/mL of EPO. Time-course analysis showed that pretreatment with EPO provided protection only for large RGCs; early post-treatment with EPO protected both small and large RGCs. Inhibitors of signal transduction and activators of transcription such as (STAT)-5, mitogen-activated protein kinases (MAPK)/extracellular-regulated kinase (ERK), and phosphatidyl inositol-3 kinase (PI3K)/Akt impaired the protective effect of EPO on RGCs exposed to different insults.

**Conclusion:**

EPO provided neuroprotection to cultured adult rat RGCs; however, the degree of protection varied with the type of toxic insult, RGC subtype, and timing of EPO treatment.

## Introduction

Glaucoma is the second leading cause of blindness in the world [1]. It is characterized by progressive death of retinal ganglion cells (RGCs) and visual field loss. Although elevated intraocular pressure (IOP) is widely accepted as the major risk factor for glaucoma, RGC death and visual field loss continue to occur in some patients with good IOP control [2]. Accumulating literature suggests that many mechanisms may be responsible for RGC death, including inflammation [3], [4], apoptosis [5], [6], excitotoxicity [7], and trophic factor withdrawal (TFW) [8], [9]. Therefore, neuroprotection, a method for preventing RGC death, has become a treatment strategy in glaucomatous optic neuropathy. Accordingly, various agents are being investigated for the treatment of glaucoma. At present, there are no FDA-approved neuroprotectants for the treatment of glaucoma. This deficiency encourages further research into the potential application of neuroprotective treatments for glaucoma. For example, an innovative approach is to use multifunctional neuroprotectants against the complex process of RGC death [10].

Erythropoietin (EPO), a glycoprotein, stimulates the production of red blood cells. Recombinant EPO is clinically used to treat anemia associated with chronic renal failure, cancer, and HIV infection. Moreover, studies have shown that both EPO and EPO receptor (EPOR) are present in the nervous system. When EPO binds to EPOR, Janus-tyrosine kinase (JAK)-2 is phosphorylated. Phosphorylation of JAK2 activates several downstream signaling pathways such as those that employ signal transducers and activators of transcription (STAT)-5, mitogen-activated protein kinases (MAPK)/extracellular-regulated kinase (ERK), and phosphatidyl inositol-3 kinase (PI3K)/Akt. These pathways can exert multiple anti-apoptotic, anti-oxidative, and neurotrophic effects to protect neurons against damage [11]. Immunohistochemistry has shown that EPOR is produced in the RGC layer, inner nuclear layer, and photoreceptor layer of the mammalian eye [12], [13]. Furthermore, rodent studies indicate that intraocular injection of EPO has neuroprotective effects in models of optic nerve transection and ocular hypertension [12]–[15]. However, it is unclear whether EPO is directly capable of moderating the effects of various insults on RGCs.

RGCs are subdivided according to morphology and function [16]. Multiple lines of evidence suggest that RGC subtypes exhibit differential responses to pathological conditions. For example, several studies indicate that elevated IOP causes selective death of large RGCs in primates [17], [18]. Other research shows that large RGCs in adult rats and cats are more resistant to axotomy than small RGCs [19], [20]. Thus, it is reasonable to assume that a neuroprotectant may have selective effects on certain types of RGCs.

The present study aimed to evaluate the neuroprotective effects of EPO on total, small, and large RGCs under *N*-methyl-d-aspartate (NMDA)-, trophic factor withdrawal (TFW)-, and tumor necrosis factor-alpha (TNF-α)-induced toxicity in *in vitro* cultures of retinal cells. The time course of neuroprotective effect exerted by EPO also was investigated.

## Methods

### Retinal Cell Culture

Animals were handled in accordance with the guidelines put forth by the Association for Research in Vision and Ophthalmology Statement on the Use of Animals in Ophthalmic and Vision Research. The study protocol was approved by the Institutional Animal Care and Use Committee of National Defense Medical Center (Permit number: IACUC-08-209). Adult rat retinal cells were obtained and cultured as previously described [21]. Briefly, adult Wistar rats were killed using CO_2_, and their eyes were quickly enucleated. Retinal cell suspensions were produced by dissecting the retinas and incubating (37°C, 30 min) in digestion buffer containing neurobasal medium (21103049; Invitrogen, Carlsbad, CA) supplemented with 2 mg/mL papain (P4762; Sigma, St. Louis, MO), 0.4 mg/mL dl-cysteine (C4022; Sigma), and 0.4 mg/mL bovine serum albumin (BSA) (A7906; Sigma). Next, retinas were washed 3 times with RGC culture medium containing 100 units/mL penicillin (P4333; Sigma), 100 µg/mL streptomycin (P4333; Sigma), 1 mM pyruvate (11360-070; Invitrogen), 2 mM glutamine (25030-081; Invitrogen), 5 µg/mL insulin (I6634; Sigma), 100 µg/mL transferrin (T1147; Sigma), 100 µg/mL BSA (A7906; Sigma), 60 ng/mL progesterone (P8773; Sigma), 16 µg/mL putrescine (P5780; Sigma), 40 ng/mL sodium selenite (S5261; Sigma), 40 ng/mL thyroxine (T1775; Sigma), 40 ng/mL triiodothyronine (T6397; Sigma), 5 µM forskolin (F6886; Sigma), 1% fetal calf serum (10437; Invitrogen), 50 ng/mL brain-derived neurotrophic factor (BDNF) (PHC7074; Invitrogen), 10 ng/mL ciliary neurotrophic factor (CNTF) (PRC7014; Invitrogen), and 10 ng/mL basic fibroblast growth factor (bFGF) (PHG0024; Invitrogen). At the end of treatment period, tissues were triturated using a disposable glass pipette to obtain a suspension of single cells. The number of retinal cells was counted using a hemocytometer (Z359629; Bright-*Line, Reichert*, *Buffalo*, NY). Retinal cells were seeded on poly-d-lysine- and laminin-coated 8-well culture slides (354108; *BD Biosciences*, Franklin Lakes, NJ) at a density of approximately 1×10^6^ cells/well with 0.5 mL/well RGC culture medium and cultured in 95% air and 5% CO_2_ at 37°C for 3 days.

### Identification of RGCs

#### Retrograde labeling of RGCs

RGCs were retrograde labeled as previously reported [22]. Briefly, rats were anesthetized using a ketamine (3542; Pfizer, Taipei, Taiwan) and xylazine (X1251; Sigma) mixture. The skin over the cranium was incised, and 2 vertical holes of 1-mm diameter were drilled on both sides of the skull at 6-mm posterior to the bregma, 1.5-mm lateral to the midline, and 4-mm depth from the bone surface by using a dentist’s drill. RGCs were labeled with Fluoro-Gold (FG) (39286; Sigma) by microinjecting 2 µL of 3% FG solution into the superior colliculi. Three days after the FG injection, rats were killed, and retinas were dissected and dissociated for culture. The presence of FG marker in the cultured RGCs was detected by fluorescence microscopy (*Olympus BX-50; Olympus* Optical, Tokyo, *Japan*).

#### Double-label immunocytochemistry

For double-label immunocytochemistry, 3-day cultures of retinal cells were fixed with 10% formalin solution (HT5012; Sigma) for 30 min. After washing 3 times with Dulbecco's phosphate-buffered saline (DPBS) (SH30264.01; Thermo Scientific *HyClone, Logan, UT*), primary antibodies (Abs), including mouse anti-Thy-1 (MAB1406; *Millipore, Bedford*, *MA,*) and rabbit anti-neurofilament-l (NF-l, 68 kDa) (AB9568; *Millipore*) (*diluted* to 1∶400 in DPBS containing 0.02% saponin [47036, Sigma]) were immunoreacted with the cells for 1 h. The cells were washed with DPBS and then incubated for 30 min with secondary Abs, including Alexa Fluor 594-labeled goat anti-mouse IgG (1∶300) (A11005; Invitrogen) and Alexa Fluor 488-labeled goat anti-rabbit IgG (1∶300) (A11008; Invitrogen), to visualize the cells labeled with primary Abs. For EPOR immunocytochemistry, primary Abs, including mouse anti-Thy-1 and goat anti-EPOR (1∶100) (E4644; Sigma), were immunoreacted with the cells. Alexa Fluor 488-labeled donkey anti-goat IgG (1∶300) (A11055; Invitrogen) was first used to detect the anti-EPOR Abs, and then Alexa Fluor 594-labeled goat anti-mouse IgG was used to detect the anti-Thy-1 Abs. Cells were washed 3 times with DPBS and then subjected to nuclear staining with 100 ng/mL DAPI solution (D8417; Sigma) for 10 min. The slide was washed with deionized water, covered with a drop of Fluoromount G (0100-01; Southern Biotech, Birmingham, AL), and coverslipped. The labeled cells were observed by fluorescence microscopy and cell soma sizes were measured.

### Toxic Insults and Drug Treatment

To induce *N*-methyl-d-aspartic acid (NMDA) toxicity, cells were cultured for 3 days in a medium supplemented with NMDA (M3262; Sigma) at 20–500 µM concentrations. To induce TFW toxicity, cells were cultured for 3 days in a medium prepared as above but without BDNF, CNTF, and bFGF. To induce inflammatory toxicity, cells were cultured for 3 days in a medium supplemented with TNF-α (PHC3013; Invitrogen) at 12.5–50 ng/mL. To investigate the effect of EPO, the culture medium was supplemented with 1, 10, or 100 ng/mL EPO (E5627; Sigma). In addition, 1–100 µM memantine (an NMDA receptor antagonist) (M9292; Sigma), 1–100 ng/mL glial cell-derived neurotrophic factor (GDNF) (PHC7041; Invitrogen), 1–10 µg/mL anti-TNF-α neutralizing Ab (AB-210-NA; R&D Systems, Minneapolis, MN), 1–10 µg/mL anti-TNFR-1 neutralizing Ab (MAB225; R&D Systems), and 1–10 µM Z-IETD-FMK (a caspase-8 inhibitor) (FMK007; Sigma) were also used as control treatments in selected cultures [5], [23]. At the time of use, hydrophilic agents were freshly prepared in deionized water, and Z-IETD-FMK was prepared in dimethyl sulfoxide (DMSO) (D2650; Sigma). The final concentration of DMSO in the culture medium was between 0.1% and 0.01% (v/v). Deionized water or DMSO was used for the respective control (vehicle) cultures. To investigate the time-course effects of EPO exposure, cells were treated with EPO at 4 and 8 h before and after the toxic insults, respectively.

### Combined Treatment with EPO and Pharmacological Inhibitor

To analyze the intracellular signaling pathways responsible for RGC survival, MAPK/ERK inhibitor (PD98059) (SC-3532A; Santa Cruz Biotechnology, Santa Cruz, CA), PI3K/Akt inhibitor (wortmannin) (SC-3505A; Santa Cruz Biotechnology), and STAT-5 inhibitor (*N*′-[(4-oxo-4H-chromen-3-yl)methylene]nicotinohydrazide) (573108; Merck, Beeston, UK) were dissolved in DMSO and added to the cultures 30 min before EPO addition.

### Assessment of RGC Survival

At the end of 3-day incubation, cells were processed for immunocytochemistry. Healthy RGCs were assessed as described previously [21], [24], [25]. Briefly, double-positive (for both Thy-1 and NF-l) cells with continuous membranes, no signs of vacuolation, no signs of perikaryal swelling, and no signs of nuclear pyknosis or framentation were quantified by manual counting.

### Statistical Analysis

All results were expressed as mean ± standard error of the mean (SEM). Statistical analyses were performed using the Student’s *t*-test or 1-way ANOVA, followed by Dunnett’s test by using the SPSS 12 software (SPSS Inc., Chicago, IL). Significant difference was set at *P*<*0.05*.

## Results

### Identification and Morphology of Adult Rat RGCs

**Figure 1:**
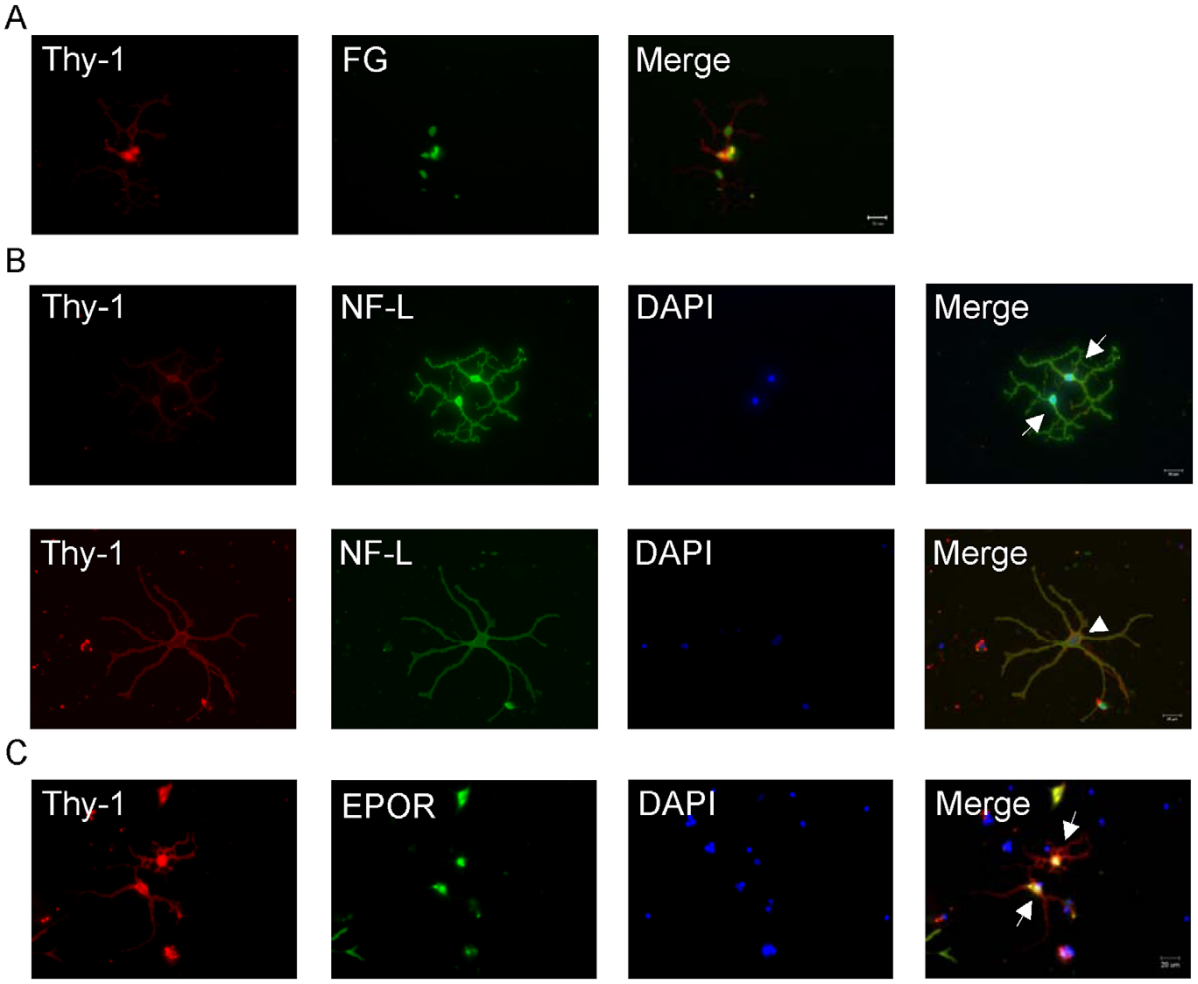
Identification and morphology of adult rat RGCs in a mixed culture. (A) Colabeling with anti-Thy-1 antibody (red) and Fluoro Gold (FG, green). Seven days after the retrograde labeling of RGCs, retinal cells were dissociated to culture, and post-culture RGCs were fixed and immunostained with anti-Thy-1 antibody. (B) Double immunocytochemistry with anti-Thy-1 antibody (red), anti-neurofilament-l (NF- l) antibody (green), and DAPI nuclear staining (blue). Images represent the 2 morphological types of adult rat RGCs: large RGCs (arrowhead) with a large soma (approximately 15–25 µm in diameter), long neurites (>80 µm in length), and large-diameter neurites and small RGCs (arrow) with a small soma (approximately 3–15 µm in diameter), short neurites (approximately 20–60 µm in length), and small-diameter neurites. (C) Double immunocytochemistry with anti-Thy-1 antibody (red), anti-erythropoietin receptor (EPOR) antibody (green), and DAPI nuclear staining (blue). Thy-1 and EPOR are coexpressed by RGCs (arrow). Scale bar = 20 µm.

In the mixed culture of retinal cells, RGCs were identified by retrograde labeling with FG, presence of specific markers, and cellular morphology. Colocalization of anti-Thy-1 Ab and FG (Fig. 1A) and anti-Thy-1 Ab, anti-NF-l Ab, and DAPI nuclear staining (Fig. 1B) were observed. Among Thy-1-positive cells, approximately 70% were also FG positive and >95% were also NF-l positive. In comparison to double immunocytochemical labeling of RGCs, FG prelabeling of RGCs identified 50%–70% cells in the cultured RGCs. Our retinal flat mount study showed that FG was prestained in rats for 3–14 days, without significant effect on RGC numbers (data not shown). This result suggests that FG may cause partial toxicity in cultured RGCs. Therefore, double immunocytochemistry would be more suitable method than FG retrograde labeling for identifying cultured RGCs, particularly to avoid false RGC counts during the labeling process. In addition, Thy-1 and EPOR immunocytostaining confirmed the presence of EPOR in RGCs (Fig. 1C). These results suggest that EPO may act on RGCs via EPOR.

Fluorescence microscopy showed that RGCs (defined as Thy-1 and NF-l double-positive cells) had ovoid soma and 2–5 primary neurite branches. These characteristics were similar to those seen in previous reports [21]. Approximately 160 cultured RGCs were seen in each 0.69-cm^2^ well. The cells were further classified into 2 types: large RGCs, which had a large soma (approximately 15–25 µm in diameter), long neurites (>80 µm in length), and large-diameter neuritis, and small RGCs, which had a small soma (approximately 3–15 µm in diameter), short neurites (approximately 20–60 µm in length), and small-diameter neurites (Fig. 1B). Microscopic observation showed that the ratio of small to large RGCs was approximately 79%–21% under our standard culture conditions.

### Neuroprotective Effects of EPO in NMDA-induced Toxicity

**Figure 2:**
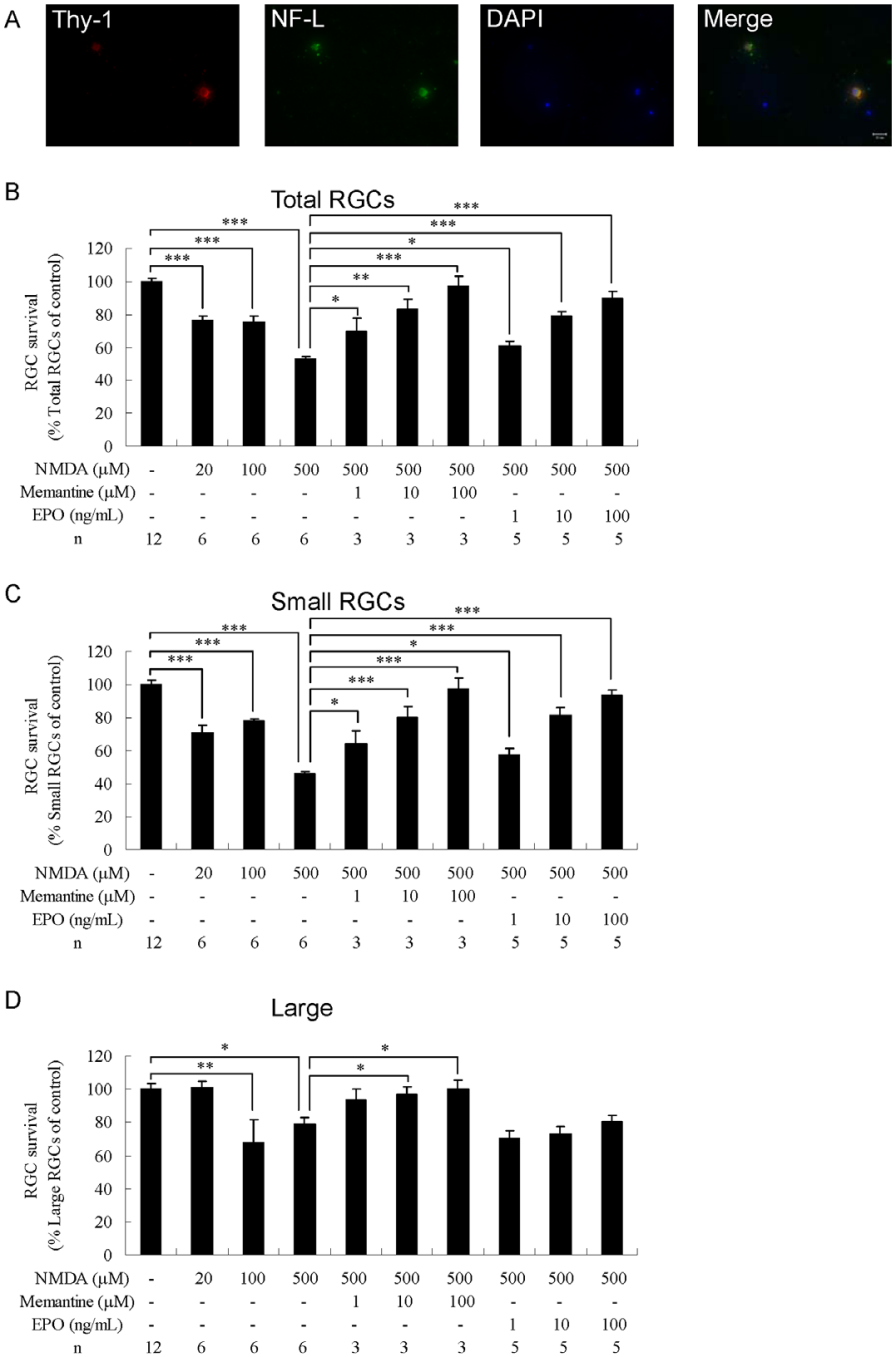
Effects of erythropoietin on total, small, and large RGCs under NMDA-induced toxicity. (A) Morphology of NMDA-treated RGCs. The cells were cultured in a medium supplemented with 500 µM NMDA. After culturing, RGCs were immunostained with anti-Thy-1 antibody (red) and anti-NF-l antibody (green), and DAPI nuclear staining (blue). Survival rate of (B) total, (C) small, and (D) large RGCs. The cells were treated with vehicle: NMDA (20–500 µM) alone, NMDA (500 µM)+memantine (1–100 µM), or NMDA (500 µM)+EPO (1–100 ng/mL). The number of RGCs was counted and normalized versus the control group. Data are presented as mean ± standard error of the mean (n = 3–12). Data were statistically analyzed by 1-way ANOVA, followed by the Dunnett’s test. *P<0.05, **P<0.01, and ***P<0.001.

To induce toxicity, RGCs from adult rats were grown in the presence of NMDA at concentrations of 20, 100, or 500 µM. After 3 days of culture, NMDA induced drastic RGC degeneration, including neurite degeneration, cell membrane discontinuity, and nuclear fragmentation (Fig. 2A). For all the 3 concentrations of NMDA, the loss or damage of total RGCs (20 µM, P<0.001; 100 µM, P<0.001; 500 µM, P<0.001; n = 6; Fig. 2B) and small RGCs (20 µM, P<0.001; 100 µM, P<0.001; 500 µM, P<0.001; n = 6; Fig. 2C) was significant compared with the control group (n = 12). At 500 µM, NMDA reduced the survival rates of total and small RGCs to 47.1±1.5% (mean ± SEM) and 53.9±1.2% that of the control group, whereas significant loss or damage of large RGCs was observed only at the intermediate (P<0.01, n = 6; Fig. 2D) and high (P<0.05, n = 6; Fig. 2D) NMDA concentrations. To evaluate whether EPO could protect small and large RGCs from NMDA toxicity, 500 µM NMDA and 1, 10, or 100 ng/mL EPO were added simultaneously to the culture medium. In addition, memantine (a known NMDA receptor antagonist) was included as a control in selected cultures. We observed that memantine (at 1–100 µM concentrations) protected total and small RGCs from NMDA-induced toxicity in a dose-dependent manner, whereas large RGCs were protected with 10 and 100 µM memantine (n = 3; Fig. 2B–D). Neuroprotection by EPO (1–100 ng/mL) of total (n = 5; Fig. 2B) and small (n = 5; Fig. 2C) RGCs was also in a dose-dependent manner, with half maximal effective concentrations (EC_50_) of 6.5 ng/mL and 5.7 ng/mL and maximal survival rates of 89.8±3.8% and 93.1±3.8%, respectively. Notably, EPO did not provide significant neuroprotection to large RGCs against NMDA-induced toxicity (n = 5; Fig. 2D).

### Neuroprotection by EPO in TFW-induced Toxicity

**Figure 3:**
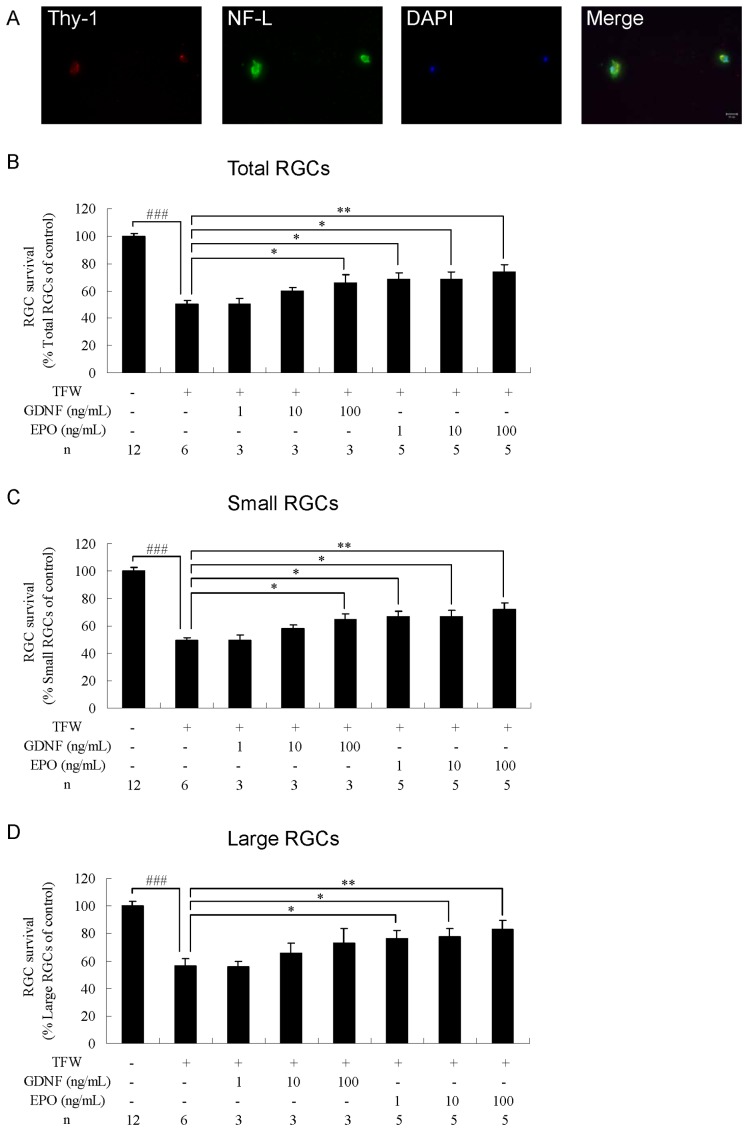
Effects of erythropoietin on total, small, and large RGCs under TFW-induced toxicity. (A) Morphology of TFW-treated RGCs. The cells were cultured in a medium lacking BDNF, CNTF, and bFGF. After culturing, RGCs were immunostained with anti-Thy-1 antibody (red), anti-NF-L antibody (green), and DAPI nuclear staining (blue). Survival rate of (B) total, (C) small, and (D) large RGCs. The cells were cultured in a standard medium, TFW medium, TFW medium+GDNF (1–100 ng/mL), or TFW medium+EPO (1–100 ng/mL). The number of RGCs was counted and normalized versus the control group. Data are presented as mean ± standard error of the mean (n = 3–12). #P<0.05, ##P<0.01, and ###P<0.001 versus the control group according to Student’s *t*-test. *P<0.05, **P<0.01 and ***P<0.001 versus the TFW group according to 1-way ANOVA, followed by Dunnett’s test.

To induce TFW toxicity, RGCs from adult rats were continuously cultured in TFW medium (RGC culture medium without brain-derived neurotrophic factor, ciliary neurotrophic factor, and basic fibroblast growth factor). After 3 days of culture, abnormal RGC morphology was observed (Fig. 3A) and survival rates of total, small, and large RGCs significantly decreased to 50.3±2.6% (P<0.001, n = 6; Fig. 3B), 49.0±4.2% (P<0.001, n = 6; Fig. 3C), and 56.2±5.4% (P<0.001, n = 6; Fig. 3D) that of the control group (n = 12), respectively. To evaluate whether EPO protected small and large RGCs from TFW toxicity, EPO (at 1, 10, or 100 ng/mL) was added to the TFW medium at the beginning of cell culture. In addition, GDNF was used as a positive control in this study. We observed that 100 ng/mL GDNF protected total and small RGCs from TFW-induced toxicity, whereas GDNF provided no significant neuroprotection to large RGCs (n = 3; Fig. 3B–D). All the 3 tested concentrations of EPO significantly increased the survival of total (1 ng/mL, P<0.05; 10 ng/mL, P<0.05; 100 ng/mL, P<0.01; n = 5; Fig. 3B), small (1 ng/mL, P<0.05; 10 ng/mL, P<0.05; 100 ng/mL, P<0.01; n = 5; Fig. 3C), and large RGCs (1 ng/mL, P<0.05; 10 ng/mL, P<0.05; 100 ng/mL, P<0.01; n = 5; Fig. 3D) under TFW-induced toxicity. At 100 ng/mL, EPO showed maximal effects, increasing the survival of total, small, and large RGCs to 73.7±5.1%, 71.9±4.9%, and 82.7±6.9%, respectively. In comparison to GDNF, EPO was more efficacious in protecting large RGCs from TFW-induced toxicity.

### Neuroprotection by EPO in TNF-α-induced Toxicity

**Figure 4:**
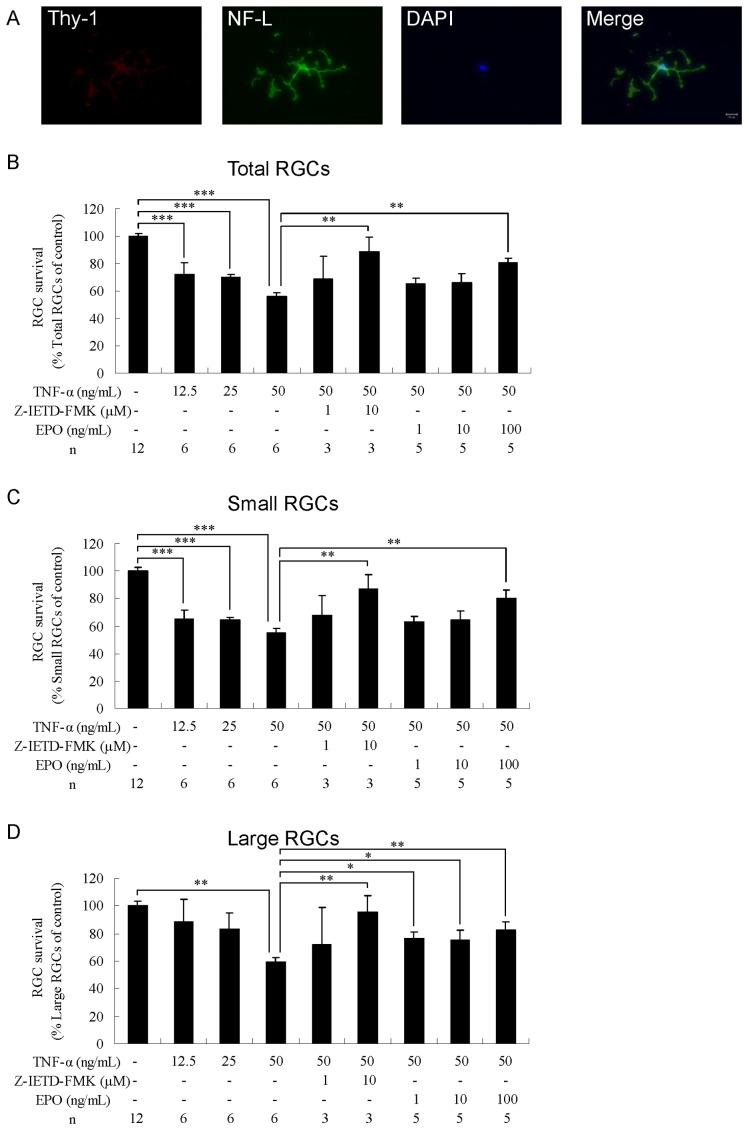
Effects of erythropoietin on total, small, and large RGCs under TNF-α-induced toxicity. (A) Morphology of TNF-α-treated RGCs. The cells were cultured in a medium supplemented with 50 ng/mL TNF-α. After culturing, RGCs were immunostained with anti-Thy-1 antibody (red), anti-NF-l antibody (green), and DAPI nuclear staining (blue). Survival rate of (B) total, (C) small, and (D) large RGCs. The cells were treated with a vehicle: TNF-α (12.5–50 ng/mL) alone, TNF-α (50 ng/mL)+Z-IETD-FMK (1 or 10 µM), or TNF-α (50 ng/mL)+EPO (1–100 ng/mL). The number of RGCs was counted and normalized versus the control group. Data are presented as mean ± standard error of the mean (n = 3–12). Data were statistically analyzed by 1-way ANOVA, followed by Dunnett’s test. *P<0.05, **P<0.01 and ***P<0.001.

To induce TNF-α toxicity, RGCs from adult rats were grown in the presence of TNF-α at concentrations 12.5, 25, or 50 ng/mL. After 3 days of culture, TNF-α exposure resulted in neurite damage in surviving RGCs (Fig. 4A). All the 3 tested concentrations of TNF-α resulted in significant loss or damage of total (12.5 ng/mL, P<0.001; 25 ng/mL, P<0.001; 50 ng/mL, P<0.001; n = 6; Fig. 4B) and small RGCs (12.5 ng/mL, P<0.001; 25 ng/mL, P<0.001; 50 ng/mL, P<0.001; n = 6; Fig. 4C) compared with the control group (n = 12). At 50 ng/mL, TNF-α reduced the survival rates of total and small RGCs to 55.7±2.8% and 55.2±3.1%, respectively of control, whereas significant loss or damage of large RGCs was observed only at the highest tested concentration of TNF-α (P<0.01, n = 6; Fig. 4D). To evaluate whether EPO protected small and large RGCs from TNF-α toxicity, 50 ng/mL TNF-α and 1, 10, or 100 ng/mL EPO were added together to the culture medium. In addition, anti-TNF-α Ab, anti-TNF receptor 1 (TNFR-1) Ab, and Z-IETD-FMK (known inhibitors of TNF-α, TNFR1, and caspase-8 activities, respectively) were included in selected control cultures. We observed that Z-IETD-FMK protected total, small, and large RGCs from TNF-α toxicity (n = 3; Fig. 4B–D). Treatment with anti-TNF-α Ab or anti-TNFR1 Ab (at 1 or 10 µg/mL) did not significantly affect RGC survival (data not shown). At 100 ng/mL, EPO increased the survival of total and small RGCs to 80.4±3.6% (P<0.01, n = 5; Fig. 4B) and 80.4±5.8% (P<0.01, n = 5; Fig. 4C), respectively. In the presence of 1, 10, and 100 ng/mL EPO, significant increases in the survival of large RGCs was observed, with rates reaching 76.5±4.5% (P<0.05), 75.2±6.9% (P<0.05), and 82.7±5.3% (P<0.01), respectively (n = 5; Fig. 4D).

### Time-course Effects of EPO on RGC Survival

To investigate the time course of EPO effects on RGC survival with NMDA, TFW, and TNF-α toxicity, RGCs were treated with EPO at 4 or 8 hr before or after the toxic insults, respectively. The results are shown in Table 1. Treatment with EPO before or after NMDA-induced injury offered protection to small RGCs. RGCs showed the highest survival rate when EPO was added at the same time as NMDA. The protective effect of EPO gradually decreased as the time of EPO addition was delayed relative to NMDA treatment. Better protection was seen when EPO was provided prophylactically (before rather than after the injury). In the TFW-induced toxicity model, treatment with EPO before injury was precluded by the need for RGCs to become adherent before RGC growth medium could be replaced with TFW medium. When adherent cells were subjected to TFW-induced damage, concomitant inclusion of EPO in the medium provided neuroprotection to both small and large RGCs. Addition of EPO at 4 h after the start of TWF-induced injury did not offer significant protective effect. When cells were subjected to TNF-α induced injury, simultaneous addition of EPO increased the survival rate of small and large RGCs. EPO treatment 4 h before the TNF-α-induced injury gave protection to large RGCs but not to small RGCs. Nonetheless, any EPO exposure, even after the injury, increased the survival rates of small and large RGCs.

**Table 1:** Time course of EPO neuroprotective effects on damaged RGCs.

	Toxic insult	RGC type	Insult only	Time of EPO administration after cytotoxic induction (h)				
				−8	−4	0	4	8
**RGC survival (%)**	NMDA	Total	52.9±1.5	69.8±4.1†	81.7±3.2‡	89.8±3.8‡	72.4±5.4†	67.2±3.4†
		Small	46.1±1.2	65.9±4.1‡	78.8±4.1‡	93.1±3.8‡	72.5±4.5‡	62.9±2.7‡
		Large	79.0±3.6	84.1±8.0	84.1±2.6	80.3±4.2	69.6±9.2	75.7±10.0
	TFW	Total	50.2±2.6	–	–	73.7±5.1†	48.6±1.7	43.1±2.4
		Small	49.0±4.2	–	–	71.9±4.9†	49.1±2.3	41.8±3.4
		Large	56.2±5.4	–	–	82.7±6.9†	47.5±2.0	46.3±2.1
	TNF-α	Total	55.7±2.8	64.5±7.8	68.4±4.5*	80.4±3.6‡	83.1±7.2†	76.7±2.7‡
		Small	55.2±3.1	65.4±9.3	65.2±3.7	80.4±5.8†	83.4±5.3†	78.4±4.3†
		Large	59.1±3.5	62.4±6.4	79.5±7.6*	82.7±5.3†	82.0±5.6†	70.7±4.8

Damage in RGCs was induced by 500 µM NMDA for 3 days, trophic factor withdrawal (removal of BDNF, CNTF and bFGF from the medium) for 3 days, or 50 ng/mL TNF-α for 3 days. (Data were normalized relative to control group [100%] and are presented as mean ± standard error of the mean (n = 6). Data were statistically analyzed by 1-way ANOVA, followed by the Dunnett’s test.

* P<0.05, †P<0.01, and ‡P<0.001 compared with insult-only groups. Classification of RGC types is described in the text.

### Effects of Pharmacological Inhibitors on RGC Survival

To analyze EPOR downstream signaling, including signaling via ERK/MAPK, PI3K/Akt, and STAT-5 pathways and associated effects on RGC survival, specific inhibitors were used to interfere with each signal transduction pathway.

### Effects of the MAPK/ERK Inhibitor on RGC Survival

**Figure 5:**
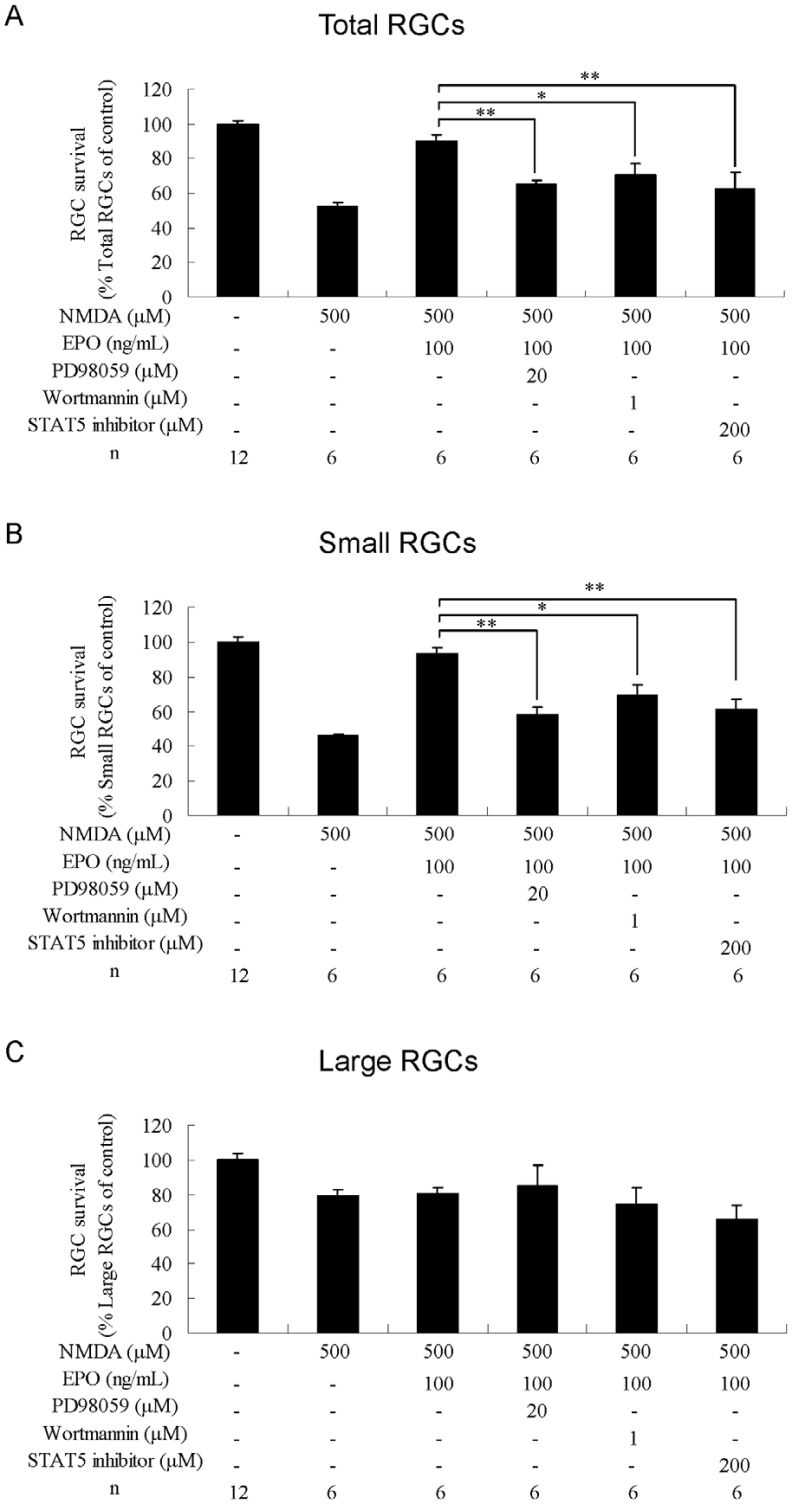
Effects of combined treatment with erythropoietin and kinase inhibitors on RGCs under NMDA-induced toxicity. Survival rates of (A) total, (B) small, and (C) large RGCs were estimated as percentages of the control. Data are presented as mean ± standard error of the mean (n = 6–12). Data were statistically analyzed by 1-way ANOVA, followed by Dunnett’s test. *P<0.05, **P<0.01 and ***P<0.001.

**Figure 6:**
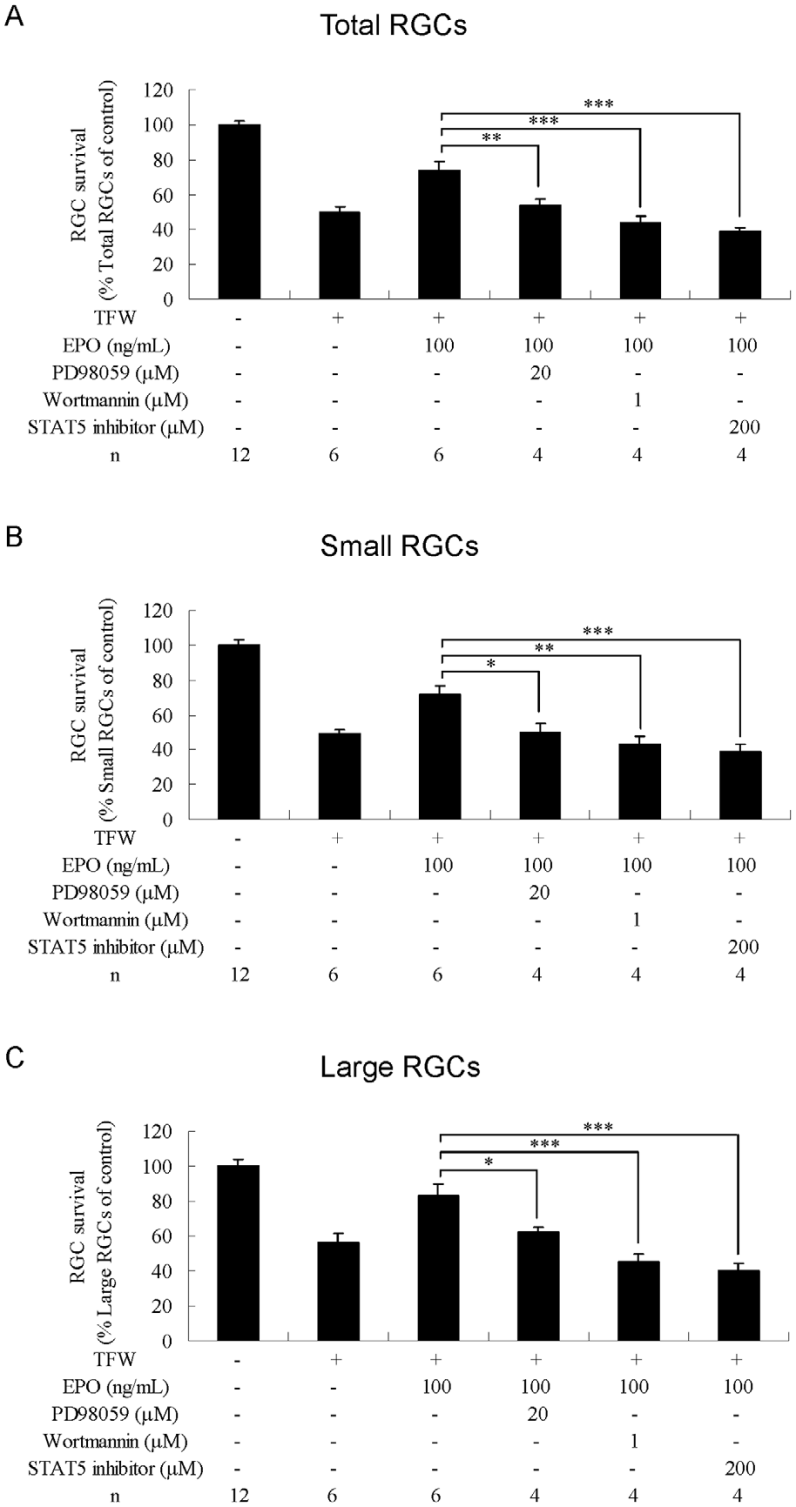
Effects of combined treatment with erythropoietin and kinase inhibitors on RGCs under TFW-induced toxicity. Survival rates of (A) total, (B) small, and (C) large RGCs were estimated as percentages of the control. Data are presented as mean ± standard error of the mean (n = 6–12). Data were statistically analyzed by 1-way ANOVA, followed by the Dunnett’s test. *P<0.05, **P<0.01 and ***P<0.001.

**Figure 7:**
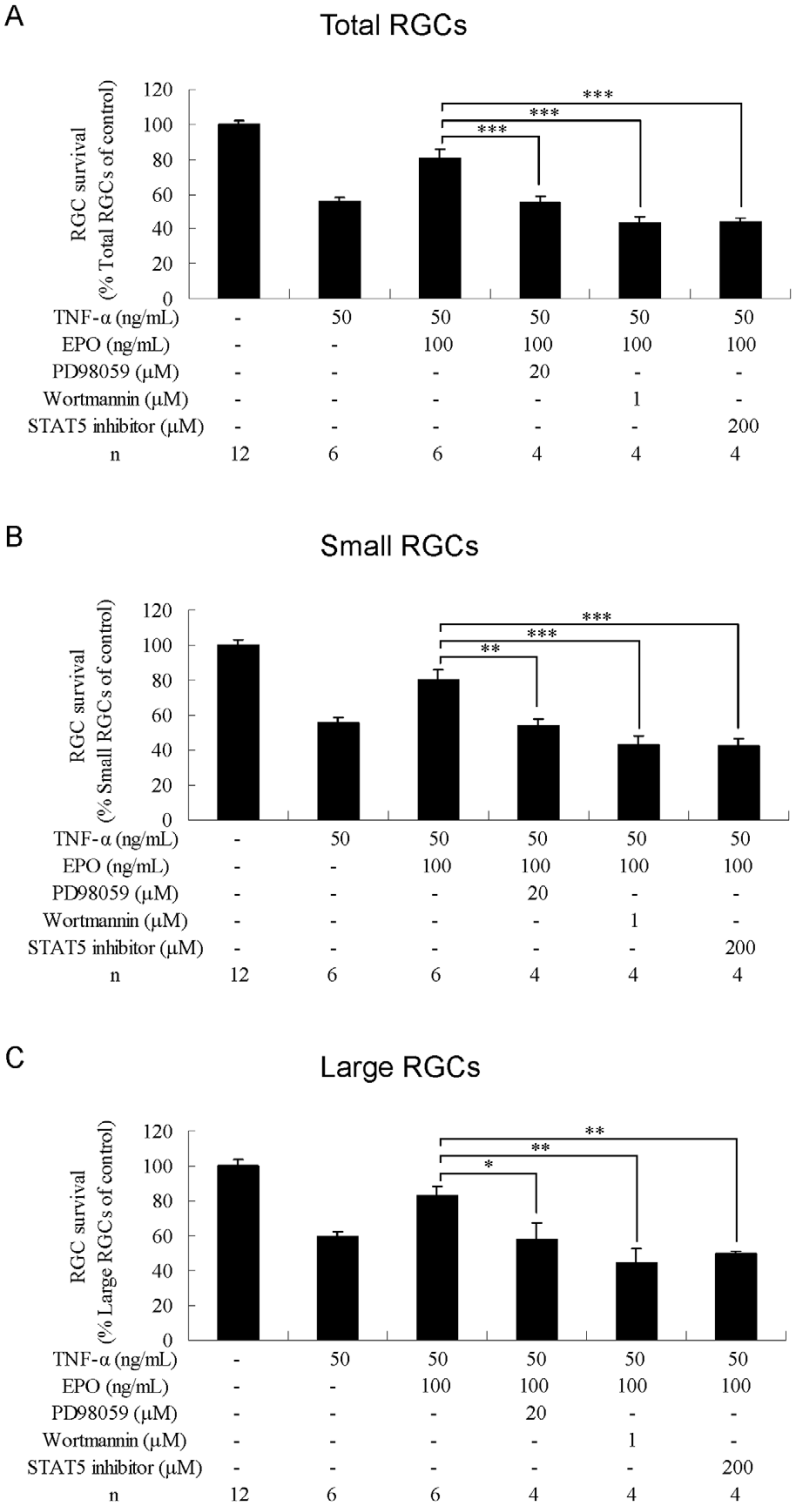
Effects of combined treatment with erythropoietin and kinase inhibitors on RGCs under TNF-α-induced toxicity. Survival rates of (A) total, (B) small, and (C) large RGCs were estimated as percentages of the control. Data are presented as mean ± standard error of the mean (n = 6–12). Data were statistically analyzed by 1-way ANOVA, followed by the Dunnett’s test. *P<0.05, **P<0.01 and ***P<0.001.

Addition of MAPK/ERK inhibitor PD98059 (20 µM) completely abolished the protective effect of EPO against NMDA-induced toxicity in total RGCs (P<0.01; Fig. 5A) and small RGCs (P<0.01; Fig. 5B) (compared with NMDA+EPO). PD98059 decreased the protective effect of EPO against TFW toxicity in total RGCs (P<0.01; Fig. 6A), small RGCs (P<0.05; Fig. 6B), and large RGCs (P<0.05; Fig. 6C) (compared with TFW+EPO). PD98059 also reduced the protective effect of EPO against TNF-α toxicity in total RGCs (P<0.001; Fig. 7A), small RGCs (P<0.01; Fig. 7B), and large RGCs (P<0.05; Fig. 7C) (compared with TNF-α+EPO).

### Effects of PI3K/Akt Inhibitor on RGC Survival

Addition of PI3K/Akt inhibitor wortmannin (1 µM) reduced the protective effect of EPO against NMDA toxicity in total RGCs (P<0.05; Fig. 5A) and small RGCs (P<0.05; Fig. 5B) (compared with NMDA+EPO). Wortmannin completely abolished the protective effect of EPO against TFW toxicity in total RGCs (P<0.001; Fig. 6A), small RGCs (P<0.01; Fig. 6B), and large RGCs (P<0.001; Fig. 6C) (compared with TFW+EPO). Wortmannin also reduced the protective effect of EPO against TNF-α toxicity in total RGCs (P<0.001; Fig. 7A), small RGCs (P<0.001; Fig. 7B), and large RGCs (P<0.01; Fig. 7C) (compared with TNF-α+EPO).

### Effects of STAT-5 Inhibitor on RGC Survival

Addition of STAT-5 inhibitor (200 µM) reduced the protective effect of EPO against NMDA toxicity in total RGCs (P<0.01; Fig. 5A) and small RGCs (P<0.01; Fig. 5B) (compared with NMDA+EPO). STAT-5 inhibitor completely abolished the protective effect of EPO against TFW in total RGCs (P<0.001; Fig. 6A), small RGCs (P<0.001; Fig. 6B), and large RGCs (P<0.001; Fig. 6C) (compared with TFW+EPO). STAT-5 inhibitor also completely abolished the protective effect of EPO against TNF-α toxicity in total RGCs (P<0.001; Fig. 7A), small RGCs (P<0.001; Fig. 7B), and large RGCs (P<0.01; Fig. 7C) (compared with TNF-α+EPO).

Taken together, these data suggest that each of these signaling pathways is required for the EPO-mediated protection of RGCs from NMDA, TFW, and TNF-α insult.

## Discussion

The present study describes the different responses of small and large adult rat RGCs to EPO under 3 apoptotic mechanisms–NMDA toxicity, trophic factor withdrawal, and inflammatory toxicity. This study also used inhibitor studies to investigate the intracellular signaling pathways responsible for RGC survival.

Many neurodegenerative diseases such as Parkinson disease, Alzheimer disease, and glaucoma are characterized by progressive death of neurons. These pathologies are usually observed in aging adults. Within the retina, different responses of adult and neonatal neurons to experimental treatment are observed. For example, several studies have shown that neonatal rat RGCs have a higher survival rate than adult rat RGCs in trophic factor-containing medium [26]. Thus, adult retinal neuron may be a more pertinent model for the evaluation of neuroprotective compounds in diseases of aging. In this study, we used primary mixed retinal culture and induced RGC injury via different conditions to assess the potential protective effects of EPO. Other research groups have used purified RGCs in their studies. In comparison to mixed retinal cell culture, such studies require a more complicated culture protocol [27]. Another model for cell culture uses RGC-5, a transformed cell line that is available in large amounts. This model allows the use of multiple analytic tools, but the characteristics of this cell line are different from those of primary RGCs [28]. A key feature of mixed retinal cell culture is the coculture of RGCs with other retinal cells, which is not compatible with commercially available cell survival analysis kits such as MTT or MTS for the analysis of RGC survival rates. We employed immunocytochemistry and RGC morphological classification to characterize RGCs; quantification was performed independently by separate researchers, ensuring internal confirmation of our results.

Rat RGCs often are morphologically classified according to their soma size, dendritic field, and branching pattern. In previous studies, various methods were used for morphological analysis, such as Nissl staining [29] and retrograde horseradish peroxidase (HRP) labeling [30]. The limitations of these methods include nonspecific labeling and incomplete RGC visualization. Similarly, although Thy-1 is widely used as a surface marker for rat RGCs, this marker also is found on macrophage and endothelial cells. The 2 types of Thy-1^+^ non-RGCs can be further distinguished by morphology or staining for NF-l antigen [31]. Moreover, some researchers have reported the use of NF-l immunostaining to visualize soma and neurites of human and adult pig RGCs [24], [32], [33]. We confirmed that NF-l is strongly expressed in the soma and neurites of cultured adult rat RGCs. Thus, the present study used Thy-1 and NF-l double immunocytochemistry to achieve specific and complete RGC visualization in rat mixed retinal cell culture. Morphologically, 3 detailed classes of rat RGCs (RG_A_, RG_B_, and RG_C_) have been defined [34], [35]. Because RG_C_ cells overlap extensively with RG_A_ and RG_B_ cells, RG_C_ cells were combined into 2 other groups, defined as large and small RGCs in the present study. Large and small RGCs are morphologically similar (respectively) to RG_A_ and RG_B_ cells previously defined in rat whole mount preparations [34], [35] and to alpha and beta cells previously defined in other species [16]. In addition, RGC groups can be classified by the use of subtype-specific markers, and this method may be easier to employ. For instance, Brn-3 immunolabeling has been used to distinguish RGC groups in mouse, cat, and monkey [36]; however, we were unable to distinguish RGC subtypes in adult rat retina by Brn-3 immunolabeling (data not shown).

Glaucoma is characterized by slow and progressive RGC loss. Several mechanisms have been proposed to explain RGC loss, such as excitotoxicity, trophic factor withdrawal, and inflammation. Accordingly, various *in vitro* and *in vivo* models that have been established to mimic RGC pathological responses are important preclinical testing tools for drug development. The model of this study is an example, which could be induced to mimic 3 types of pathological responses for evaluating neuroprotectants. Unfortunately, the model is not a perfect mimic of the human disease. The duration of the model shows the lack correlation to human condition. The progression of glaucoma in humans may take years, whereas RGC loss in the present model appeared within 3 days. It is probable that these molecular events are responsible for partial stages of glaucoma. NMDA exposure is a model of acute toxicity for the retina and is often used to assess neuroprotective activity. In this *in vitro* model, we showed that NMDA was toxic for the majority of small RGCs and that treatment with EPO (at 1–100 ng/mL) ameliorated NMDA-induced killing. The large RGCs were more resistant to NMDA toxicity; therefore, the EPO treatment did not significantly alter the survival rate of this RGC subtype. The differential response of small and large RGCs also was observed in BDNF studies [37] and our pervious animal studies [22], and the underlying mechanisms require further research. In particular, our data showed that EPO treatment before NMDA injury is more beneficial than post-injury treatment. This prophylactic effect may reflect the quick mode of action of NMDA toxicity, as pointed out in a previous study where the intravitreal injection of 200 nmol NMDA in rat eyes resulted in 20% RGC death within 6 h after injection [38]. Treatment with EPO presumably requires some time to activate cell survival signals; therefore, activation of these signals before the initiation of NMDA apoptotic signals would have a beneficial protective effect. Binding of TNF-α to TNFR-1 can activate caspase-related signals leading to cell death as well as activation of NF-κB-related cell survival pathways [39]. Our data suggested that Abs against TNF-α or TNFR-1 do not effectively suppress TNF-α-induced injury in RGCs. However, inhibition of caspase-8 (downstream of TNFR-1) could abolish TNF-α toxicity to RGCs. These results may reflect simultaneous activation of RGC survival signal via NF-κB through the TNFR-1 pathway, as described in a previous study [5]. EPO is known to activate NF-κB signaling and reduce TNF-α-induced cell damage [40], [41]. Our research shows that EPO can reduce the damage in RGCs caused by TNF-α. Notably, large RGCs were more sensitive to EPO protection than small RGCs, but small RGCs were more sensitive to TNF-α toxicity than large RGCs. In addition, prophylactic administration of EPO protected large RGCs but not small RGCs. We speculate that because of the instability of EPO, the agent may lose its activity in the culture medium over time. In the TFW model, 3 types of trophic factors (BDNF, CNTF, and bFGF) were removed, thus reducing survival rates for both small and large RGCs. EPO treatment attenuated TFW-mediated toxicity in both RGC types. Previous histological analysis showed that high IOP selectively damages large RGCs. It is possible that large RGCs have a smaller surface-to-volume ratio [17], [18], [42]. Taken together, our data and the data obtained from models suggest that these differential insults affect small and large RGCs in distinct ways. Analysis of the response of small and large RGCs to EPO will be required to understand potential clinical application of EPO as a neuroprotectant. Furthermore, some neuroprotectants that have a single mode action may not have significant beneficial effects on toxic damage in other RGCs; thus, understanding the neuroprotectant’s protective spectrum is helpful for slowing RGC loss. This study showed that EPO (1–100 ng/mL) and memantine (1–100 µM) exhibited dose-dependent neuroprotection against NMDA toxicity. In TFW-induced damage model, 1, 10, and 100 ng/mL EPO concentrations exhibited RGC neuroprotection. Although GDNF groups seem to have a dose-dependent tendency, our results showed 1 and 10 ng/mL GDNF did not have a significant effect. The results indicate that higher dose of GDNF is necessary to provide protection in RGC. In the TNF-α-induced damage model, significant RGC protection occurred only at the highest tested concentration of EPO (100 ng/mL). These results showed the degree of EPO protection varied with the type of toxic insult. It is possible that EPO moderates cell death signals induced by various toxic insults through the activation of different cell survival signals. However, the inference awaits further investigation.

The protective effects of EPO may result from the activation of known signal transduction pathways such as those involving STAT-5, MAPK/ERK, or PI3K/Akt [11], [43]. Our cultured retinal cell model showed that inhibition of any of these 3 pathways could reduce the protective effect of EPO in small and large RGCs. Previous studies showed that EPO offered protection to retina; however, several different signaling pathways are proposed to mediate this effect [12], [44]–[46]. Although the details are not completely understood, several possible reasons can be proposed for the differences. First, the disparities could be attributed to differences in the species of animal models. For example, in rats, EPO is known to signal via PI3K/Akt and not via the ERK pathway to protect axotomized RGCs [12], which is the opposite of the case in mice [44]. Second, the disparities could be attributed to differences in injury models. For example, the previous demonstration of EPO protection of retinal cells (via the ERK pathway) was performed in diabetic rats [45] in contrast to the *in vitro* models used in the present work. Third, the disparities could be attributed to the effects of other growth factors such as BDNF and VEGF. The protective effects of these trophic factors on RGCs also involve these signal transduction pathways [47], [48]. Fourth, the disparities could be attributed to the differences in methodology. Notably, changes in protein levels are often analyzed by immunoblotting. However, such an analysis would only describe molecular changes for the whole retina, potentially obscuring information for individual retinal cell types.

This study showed that treatment with EPO at 1–100 ng/mL offers different levels of protection to small and large RGCs exposed to different toxicities. In particular, the highest EPO dose level offered the most complete protection to all the RGCs. The physiological EPO concentration in the human vitreous humor is estimated to be 11–460 mU/mL (equivalent to 0.11–4.6 ng/mL) [49]–[51]. To expose RGCs to a concentration of EPO similar to that tested in this study, exogenous EPO supplement would be required. Because EPO in the circulation is able to cross the blood-retina barrier into the eye, systemic administration of EPO could increase EPO concentration in the eye. However, administration of EPO via the parenteral route is expected to require high dose levels (>3000 U/kg) to provide neuroprotection [13]; such high dosages with EPO would result in unwanted side effects such as angiogenesis or carcinogenesis [52]. In addition, circulating EPO is rapidly cleared by the liver and kidney, with a half-life of approximately 3 h. Therefore, repeated injections would be necessary to maintain the effective levels. In contrast, because the eye is regarded as a closed system, local administration could reduce hepatic and renal clearance. In our cell culture model, EPO showed neuroprotection for up to 3 days. Previous studies in rabbits showed that intravitreal injection could be used to increase EPO concentration locally in the eye, with a longer half-life (approximately 3 days) [53]. Although intravitreal injection could reduce the required dose and systemic clearance of EPO, it would not provide long-term neuroprotection. Developments in targeted drug delivery or controlled drug release systems will be needed to address these obstacles [52].

In conclusion, large RGCs were more resistant to NMDA toxicity than small RGCs, and EPO treatment significantly increased the survival rate of small RGCs, especially when the treatment was initiated prophylactically. Under the influence of TFW toxicity, EPO offered protection to both small and large RGCs. Protection of large and small RGCs against TNF-α toxicity was seen at EPO concentrations of 10 ng/mL and 100 ng/mL, respectively. For both the RGC subtypes, protection required EPO treatment (even at the highest tested concentration) to start within 4 h of TNF-α exposure. Inhibitor studies suggested that the protective effects of EPO may be mediated via the activation of 1 or more signal transduction pathways, including those involving STAT-5, MAPK/ERK, or PI3K/Akt.
